# A Systematic Review of Research on Autism Spectrum Disorders in Sub-Saharan Africa

**DOI:** 10.1155/2016/3501910

**Published:** 2016-10-30

**Authors:** Amina Abubakar, Derrick Ssewanyana, Charles R. Newton

**Affiliations:** ^1^Kenya Medical Research Institute-Wellcome Trust Collaborative Programme, Kilifi, Kenya; ^2^Department of Psychiatry, University of Oxford, Oxford, UK; ^3^Department of Public Health, Pwani University, Kilifi, Kenya

## Abstract

The burden of autism spectrum disorders (ASDs) in sub-Saharan Africa (SSA) is not well known. We carried out a systematic review of the literature to identify published work from SSA. We have systematically searched four databases, namely, Medline, PsycINFO, CINAHL, and Child Development & Adolescent Studies, through EBSCO and identified studies from across SSA. Based on predefined inclusion criteria, 47 studies were included in this review. Most of the identified studies (74%) were conducted in only 2 African countries, that is, South Africa and Nigeria. Additionally, most of these studies (83%) were carried out in the last decade. These studies had four major themes: development of measurement tools of ASD in Africa, examining the prevalence of ASD, identifying risk factors and risk markers, and examining psychosocial issues. We identified only a single population level study aimed at documenting the prevalence of ASD and could not identify a single case-control study aimed at examining a comprehensive set of potential risk factors. All intervention studies were based on very small sample sizes. Put together, our findings suggest that current evidence base is too scanty to provide the required information to plan adequately for effective intervention strategies for children with ASD in Africa.

## 1. Introduction

Autism spectrum disorders (ASDs) are a neurodevelopmental syndrome with growing global health concern. This syndrome is characterized by deficits in social and communication skills and restricted and repetitive behaviour; and these adversely impact quality of life of those affected as well as their families [1]. Globally, one in every 160 persons is estimated to live with ASD, contributing to 7.6 million disability life adjusted years [2]. However, this burden is currently underestimated since prevalence of ASD in the African region and other low or middle income regions is still unclear [2–4]. One study, for example, that involved two North African countries documented a high frequency of ASD at 11.5% and 33.6% among African children with developmental disorders [5]. Other studies conducted among children of African descent have reported high occurrence of ASD [6–8] although their representativeness is questionable [9]. Similarly, studies on ASD document a large burden of nonverbal ASD cases (50−71%) and over 60% comorbid intellectual disability among African children with ASD [10, 11]. These and other distinctive traits of ASD in Africa such as a potential infectious aetiology, late diagnosis, and poor management [2, 3, 12] accentuate the need for more research focus and public health response in this region.

Having gone past the prior dialogue questioning the universality of ASD [13], growing interest in ASD in Africa is currently documented by the increasing number of scientific studies on this condition in the continent [4, 9]. There have been a few reviews synthesizing the data on ASD from Africa [4, 9]; however, most of them were performed years ago, used a single bibliographic search, or did not utilize a systematic review. Although these earlier reviews provide interesting insights, there is a need to update and synthesize the most recent empirical evidence so as to identify research gaps and potential points of interventions. Of major interest is a clearer understanding of the current direction of ASD research in Africa (e.g., the focus on risk factors, prevalence, or interventions), identifying where in Africa ASD research is emanating as well as the key findings from ASD research in the African region. This current systematic review builds upon this backdrop by exploring ASD research in the sub-Saharan African (SSA) region over the past 50 years. We hope that this systematic review will avail relevant evidence to support and guide research, intervention, and policy on ASD, especially in the SSA region.

## 2. Methods

### 2.1. Search Strategy

Guidelines for preferred reporting items for systematic reviews and meta-analyses (PRISMA) were utilized [58]. We searched four databases, that is, Medline (1935 to June 2016), PsycINFO (1935 to June 2016), CINAHL (1935 to June 2016), and Child Development & Adolescent Studies (1935 to June 2016), through EBSCO. The search terms used were “Autism” OR “Autistic” OR “Pervasive” AND “Africa”. Our database search was restricted to peer-reviewed articles and excluded dissertations. We further searched the reference lists of retrieved articles as well as the Google Scholar database for other potentially relevant studies that may have been missed from the systematic database search.

### 2.2. Criteria for Inclusion and Exclusion

We set out the following inclusion criteria:

1. The study must be an empirical study on ASD conducted among humans.
2. ASD must be the main condition of study.
3. The study populations need to be from SSA and the study needs to be carried out in SSA.

We excluded studies that (i) were not empirical, (ii) were conducted in countries other than those from SSA, and (iii) did not consider ASD as the main disorder of interest.

### 2.3. Data Extraction and Analysis

One data extraction sheet was used to summarize the data in Microsoft Excel spreadsheet (version 2013) on the general characteristics of the studies and their key findings. These characteristics of interest included (i) author, (ii) year of publication, (iii) country where the study was done, (iv) sample description, and (v) key findings. A narrative synthesis was used to summarize the findings of eligible studies included in this systematic review.

## 3. Results

### 3.1. Summary of Study Characteristics

**Figure 1:**
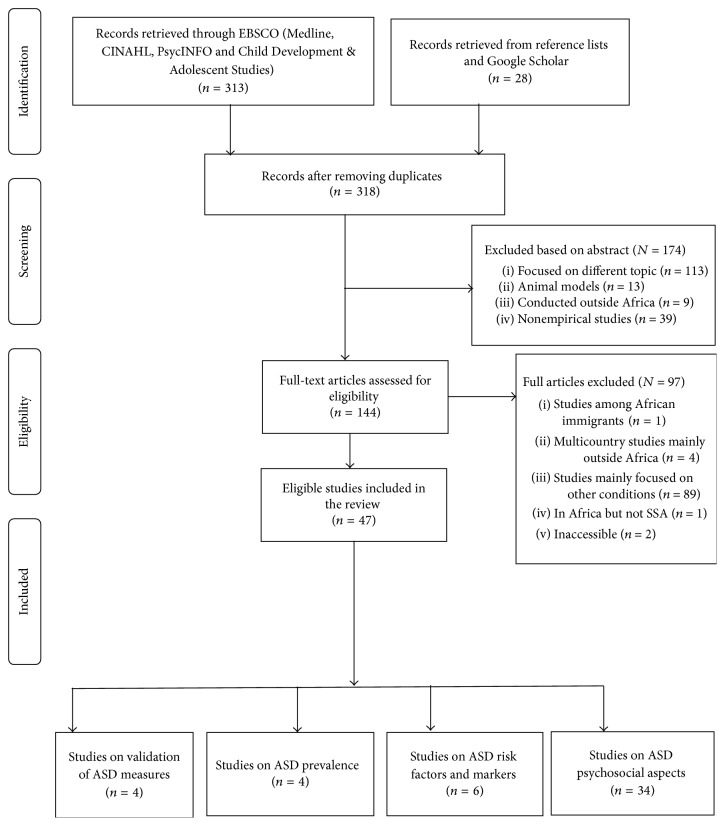
A flow diagram of ASD study selection for the systematic review.

We identified a total of 341 potentially eligible studies of which 47 fulfilled the criteria.  Figure 1 presents the flow chart on the number of identified abstracts, reasons for exclusion, and articles that were further considered.  Table 1 presents a summary of the characteristics of the eligible studies and their key findings. These eligible studies were conducted in very few African countries (around ten). Most of these studies were from South Africa (*n* = 25, 51%) and Nigeria (*n* = 11, 23%). Most of the studies (*n* = 38, 83%) were carried out in the past decade indicating increased interest in the area.

### 3.2. Screening and Diagnosis of ASD in Africa

There are few studies that have attempted to validate screening and diagnostic measures for use in SSA. A recent study from Uganda adapted and extended the Ten-Question Questionnaire (TQQ) into a 23-item questionnaire and evaluated the sensitivity and specificity of this new tool to identify ASD alongside general “disability” [17]. It was reported that the 23-item questionnaire was modestly successful in identifying a subgroup of children at high risk of being diagnosed as having ASD. A study from Tanzania evaluated the potential use of the Childhood Autism Rating Scale to perform a structured observation to diagnose ASD [16]. In this study, the Childhood Autism Rating Scale was culturally adapted for use in Tanzania. Some of the cultural adaptations included ensuring that the play interactions, materials used, and social routines used to probe the child's behaviour were familiar to the children. Following these adaptations, the authors reported excellent discriminative validity and acceptable levels of sensitivity and specificity. Two recent studies in South Africa have also examined and evaluated the cultural adaptability of ASD measures in their context [14, 15]. The study by Smith and colleagues evaluated the cultural appropriateness of the Autism Diagnostic Observation Schedule-2 (ADOS-2) [15]. Participants were requested to evaluate the cultural appropriateness of the materials and procedures for administering the ADOS. They reported that most of the social interaction demands, materials, and activities were appropriate for use in the urban samples from Cape Town. However, potential linguistic and semantic biases were observed and therefore guidelines for using ADOS in their setting were developed.

### 3.3. Prevalence of ASD

A few studies have attempted to estimate the burden of ASD in SSA [17, 19]. However, most of these studies used convenience sampling with data largely from hospital and specialist units for children with special needs. For instance, a study by Lagunju and colleagues [18] recruited 2320 patients at a paediatric neurological clinic. After a systematic screening, 54 of the 2320 patients were diagnosed with ASD, with estimated prevalence of 2.3%. Additionally, it was noted that parents reported a deviation in development at a mean age of 22.1 months, and they received a diagnosis at a mean age of 44.7 months. Among those with ASD, approximately 75.5% presented with associated neurological comorbidities. Only one community-based study was identified, in which 1169 Ugandan children aged 2–9 years were surveyed in the Kampala District (half urban and half rural) and eight children had a positive diagnosis of ASD. The authors reported unadjusted prevalence for ASD of 6.8/1000 [17]. All the studies reported higher prevalence among boys compared to girls. Bakare et al. [19] in a study from Nigeria reported an ASD ratio of 4 : 1 for boys and girls, respectively.

### 3.4. Risk Markers and Risk Factors for ASD

We did not identify any case-control study that examined a comprehensive set of risk factors for ASD in SSA. Small studies have identified specific genetic risk markers and nongenetic risk factors for ASD [11, 21]. Infectious diseases such as falciparum malaria have been suggested as possible antecedents to ASD [11], but the association has not been established. Of the six studies identified in this category, 4 explored possible genetic and biomedical factors [11, 20–22]. Three of the studies looked at potential genetic markers, the ones by Ezegwui et al. [20], Sharma et al. [21], and Arieff et al. [22]. The studies observed that certain genetic characteristics, for example, allele and genotype frequencies of 5-HTTLPR, were more likely to be associated with an increased risk of ASD. Psychosocial risk factors such as parental stress level have been associated with increased risk of ASD. Unfortunately, the study had a very limited sample size (*n* = 2) compromising the generalisability of these findings [24].

### 3.5. Psychosocial Aspects of ASD

This category presents the bulk of studies on ASD in Africa. These studies largely examined (a) awareness levels, (b) quality of services provided to children with ASD and caregiver challenges, and (c) sociocultural aspects around ASD, for example, explanatory models on the aetiology of ASD. For instance, most of the healthcare workers in Enugu, Nigeria, had limited knowledge of ASD and perceived the quality of healthcare provided to families of children with ASD as suboptimal [50, 51]. Most of the studies on awareness were carried out in Nigeria by Bakare and colleagues [33, 48, 50, 51]. One South African study observed that there were relatively few barriers to participating in ASD research and suggested that most of these barriers were poverty-related [42]. Additionally, a few very-small-scale studies (with participants ranging from 1 to 7) have looked at potential intervention strategies to enhance outcomes among children with ASD [59, 60]. For instance, Travis and Geiger investigated the efficacy of the Picture Exchange Communication System (PECS) to improve communication skills in two South African children with ASD [59]. In Tanzania, Harrison and colleagues [27] recently developed an intervention to raise awareness and help caregivers learn some basic behavioural intervention strategies in two phases. In the first phase, 14 caregivers took part in a needs assessment session and an ASD knowledge intervention. In the second phase, 29 caregivers were involved in an intervention focusing on basic behavioural strategies such as parenting skill training, teaching of basic skills (e.g., making eye contact and imitating), and teaching of self-help skills (e.g., feeding). Initial evaluation indicated that despite a few challenges it was feasible to implement the intervention and almost all participating caregivers found it to be useful.

## 4. Discussion

We reviewed the published literature on ASD in sub-Saharan Africa. Our results indicate that there is very limited data from Africa compared to other parts of the world. We identified only a single population level study aimed at documenting the prevalence of ASD in Africa. Additionally, we could not identify a single case-control study aimed at examining a comprehensive set of potential risk factors in Africa. Also, the few intervention studies had very limited sample sizes, were largely cross-sectional, and lack any measurement or evaluation of long-term impact. Put together, our findings suggest that current evidence is too scanty to provide the required information to plan adequately for effective intervention strategies for children with ASD in SSA.

Notable is the fact that most of the identified studies arose from only two African countries, that is, South Africa and Nigeria. The lack of literature from other parts of Africa may be due to several factors. First, there may be a lack of expertise in other African countries; for instance, a recent conference report does document the large difference in number of qualified psychologists and psychiatrists in South Africa compared to other countries that had representatives [61]. Second, this may arise from the lack of resources to carry out research in this area. Another potential explanation is the lack of interest in ASD as a research topic in other parts of Africa. Without any evidence, we can only speculate on the potential reasons for this. However, we feel that the dearth of research from the other African countries reflects the interaction between the first two reasons mentioned earlier. This calls for an urgent need to develop capacity and interest in ASD research in other countries outside SA and Nigeria to expand the evidence base.

An important step towards having an adequate research framework is to have standardized tools for screening and diagnosing. Our research indicates that they are very few tools that have been validated for the African context. The use of tools from other continents does provide challenges for various reasons including methodological and resource availability [15, 61]. These challenges are the impetus towards the development and validation of tools in the African context. However, some studies [16, 17] present early, yet very crucial steps towards identification of potential screening and diagnostic tools for the African context. The need to invest more into this process cannot be overemphasized given the potential benefit in accessing tools that can contribute towards early identification of children who have ASD.

Our review indicates that a significant proportion of the studies were on psychosocial issues. A major focus of most studies on psychosocial aspects of ASD potentially reflects significant burden on quality of life that African communities are increasingly witnessing and appreciating as resultant from the ASD condition. Thus, there is growing interest in understanding better care and management practices to avert this burden.

### 4.1. Limitations

We have systematically searched several databases and identified studies from across different settings in Africa. We did not look at grey literature and other more local and regional based databases; consequently, we may have missed out some articles. We, however, did carry out a search of references list to identify more studies. We hope this reduces the potential number of missed studies; however, we cannot completely exclude this possibility.

### 4.2. Conclusions

Based on our review of published works, it can be said that there is a dearth of scientifically vigorous published work from sub-Saharan Africa making it difficult to estimate the burden of ASD in this population, identify risk factors, or even plan effective intervention strategies.

## Figures and Tables

**Table 1:** A summary of empirical studies from sub-Saharan Africa.

	First author	Year	Country	Sample description	Summary of results
*Adaptation and validation of screening and diagnostic tools*					

1	Chambers [14]	2016	South Africa	26 children (15 children with no reported developmental concerns and 11 referred for suspected autism spectrum disorder)	Several measures such as early screening for autism and communication disorders and the systematic observation of red flags were translated, adapted, and evaluated for potential use among isiZulu speakers in South Africa. It was observed that children with autism presented with significantly more red flags than those without ASD. According to the authors, these results provided initial evidence that the measures are feasible for use in isiZulu speakers in South Africa.

2	Smith [15]	2016	South Africa	47 children and their caregivers	The study examined the potential impact of cultural factors on the use of Autism Diagnostic Observation Schedule-2 administration in South Africa. The authors noted potential linguistic and semantic related biases which led to the development of guidelines for using ADOS in their setting.

3	Harrison [16]	2014	Tanzania	41 children referred to psychiatric clinics	Initial results indicated that an observational approach utilizing the Childhood Autism Rating Scales may present a potentially sensitive approach to autism diagnosis in an African setting.

4a	Kakooza-Mwesige [17]	2013	Uganda	1169 children in a community survey	The study modified and extended the Ten-Question Questionnaire so as to be used to screen for ASD and other neurodevelopmental disorders in Uganda.

*Prevalence*					

5	Lagunju [18]	2014	Nigeria	2,320 patients were seen at a paediatric neurological clinic and 54 of them had ASD	The study reported 2.3% ASD prevalence.

4b	Kakooza-Mwesige [17]	2013	Uganda	1169 children in a community survey	An unadjusted prevalence rate of 6.8/1000 was reported.

6	Bakare [19]	2012	Nigeria	44 children had intellectual disability and 5 of them had ASD	Five (11.4%) of the children studied met the diagnostic criteria for childhood autism. Male/female ratio was 4 : 1.

7	Lotter [6]	1978	6 African countries	1312 were surveyed at psychiatric hospitals, schools for children with special needs, daycare centres, and homes for motherless babies and 30 met the criteria for ASD	The study observed quite a number of similarities in the presentation of ASD in African children compared to British children (e.g., higher prevalence among boys and its existence across a wide range of IQ). Additionally, the authors also observed marked differences in the prevalence of certain symptoms such as lower rates in occurrence of ritualistic and repetitive behaviour.

*Risk factors and markers*					

8	Ezegwui [20]	2014	Nigeria	21 children with ASD	Significant refractive error, mainly astigmatism, was noted in the children with autism.

9	Sharma [21]	2013	South Africa	136 children with ASD and 208 controls	The aim of the study was to find the genetic association of intronic rs736707 and exonic rs362691 (single-nucleotide polymorphisms [SNPs] of the RELN gene) with autism in a SA population. A significant association of SNP rs736707, but not for SNP rs362691, with autism in the SA population was observed.

10	Arieff [22]	2010	South Africa	109 children with ASD	Allele frequencies and genotypes of the South African autistic populations (African, mixed, and Caucasian) were compared with matching South African ethnic control populations. The study showed significant differences in allele and genotype frequencies of 5-HTTLPR polymorphic region and provides impetus for investigating the role of transmission of the L and S alleles in families with autism in South Africa.

11	Bakare [23]	2008	Nigeria	One child with ASD	Observed ASD in a child with oculocutaneous albinism.

12	Claassen [24]	2008	South Africa	A pair of twin siblings (1 had infantile autism; the other is a control)	Using data from a dizygotic set of twins, the author concluded that prenatal stress may be a salient contributor to the pathogenesis of autism.

13	Mankoski [11]	2006	Tanzania	20 children recruited from a special needs primary school and from families having a child with clinically diagnosed autism	The study aimed at investigating the association between malaria and autism. Using a case series approach, the study observed 14 children who met the criteria for ASD among whom 3 had onset of autism after recovery from malaria and 4 cases had a temporal relationship between ASD and malaria though it seemed spurious, while in seven cases the onset of ASD was unrelated to malaria.

*Psychosocial aspects*					

14	Schlebusch [25]	2016	South Africa	180 families of children with ASD	In families where there was higher frequency of regular family routines, there was also a higher satisfaction level of family quality of life (FQOL). Moreover, the cognitive appraisal of impact of ASD mediated the relationship between family routines and FQOL.

15	Tilahun [26]	2016	Ethiopia	Participants comprised caregivers (*n* = 102) of children with developmental disorders: 66.7% (*n* = 68) had a diagnosis of intellectual disability while 34 children (33.3%) had ASD as their primary diagnosis	Stigma was commonly reported (43.1%) by participants. Moreover, a significant number were ashamed of their children and some made an effort to keep their children's condition a secret. Caregivers gave a mixture of biomedical explanations (e.g., head injury (30.4%) or birth complications (25.5%)) and supernatural explanations (e.g., spirit possession (40.2%) or sinful act (27.5%)) for their child's condition. The biggest reported unmet need was educational provision for their child (74.5%), followed by treatment by a health professional (47.1%). Many caregivers also used support from friends (76.5%) and prayer (57.8%) as coping mechanisms.

16	Harrison [27]	2016	Tanzania	44 Tanzanian families of children diagnosed with ASD or general developmental delays (12 families in phase I and 29 in phase II)	The study focuses on the development of an intervention designed to inform parents about ASD and empirically supported behavioural strategies.

17	Majoko [28]	2016	Zimbabwe	21 regular teachers with experience in teaching children with ASD	This study examined barriers to including children with ASD in mainstream classes in Zimbabwe. Some of the key barriers were social rejection, communication impairments, and behavioural challenges of children with ASD. The authors proposed further training for regular teachers, collaborations with stakeholders, and enhanced social support services as strategies for encouraging inclusion of children with ASD.

18	Gona [29]	2016	Kenya	103 participants (60 parents of children with ASD and 43 professionals)	The study examined the challenges and coping strategies of parents of children with ASD. Some of the common challenges included stigma, lack of appropriate health services, and financial and heavy caregiver burden. Coping strategies applied by parents comprised problem-focused aspects that involve diet management and respite care and emotion-focused aspects that consist of beliefs in supernatural powers, prayers, and spiritual healing.

19	Meiring [30]	2016	South Africa	14 (7 parents and 7 professionals)	The study examined some of the challenges experienced by adolescents transitioning into adulthood highlighting some of the challenges such as lack of planning and the absence of service facilities for adolescents with autism after school. Other issues arising from the study included feelings of fear and uncertainty. It was also noted that this was a challenging and stressful time for parents and professionals alike.

20	Van Biljon [31]	2015	South Africa	Retrospective review of 141 children diagnosed with ASD attending a special needs school comparing two periods: 1992–2002 and 2003–2014	No significant differences in age of onset of autistic symptoms, diagnosis, sex ratio, and person who referred the child to the school. More children were likely to attend nursery schools prior to starting at the special needs school.

21	Pileggi [32]	2015	South Africa	93 children with typical developing, intellectual disability, and ASD	The researchers investigated the side of cradling infants and observed that typically developing children and children with intellectual disability had a bias towards left side cradling while children with ASD did not have this bias. They attributed the lack of left side cradling bias in children with ASD to deficits in social-affective attachment.

22	Bakare [33]	2015	Nigeria	757 final year medical students	These students had high mean scores indicating a fairly good knowledge of ASD although there were still important knowledge gaps observed.

23	Gona [34]	2015	Kenya	103 parents of children with ASD, special needs teachers, clinicians, and social workers	Preternatural causes were mentioned and included evil spirits, witchcraft, and curses. Biomedical causes comprised infections, drug abuse, birth complications, malnutrition, and genetic related problems. Treatment varied from traditional and spiritual healing to modern treatment in health facilities and included consultations with traditional healers, offering prayers to God, and visits to hospitals.

24	Eseigbe [35]	2015	Nigeria	167 medical doctors	The study aimed at evaluating the knowledge of ASD among healthcare providers and identifying challenges associated with its management. This was done using a self-administered tool, the Knowledge about Childhood Autism among Health Workers (KCAHW) questionnaire. It was observed that paediatricians and psychiatrists had a better knowledge of ASD. The highest knowledge gap was associated with onset of ASD and its comorbidities while the least one was concerning communication impairments. Some of the major challenges encountered in ASD management were the dearth of specialist services, cost of evaluation, and poor caregiver perspectives of ASD.

25	Mitchell [36]	2014	South Africa	7 parents of children with ASD	The study highlights the difficulties parents face in getting their children diagnosed with ASD partly due to the reluctance of professionals to label children as having ASD.

26	Hoogenhout [37]	2014	South Africa	86 children with ASD	The study observed that children who experienced high-functioning ASD, Asperger's syndrome, and PDD-NOS displayed delayed Theory of Mind (ToM) onset compared to a typically developing group (*n* = 30), but normal ToM developmental rates and sequences.

27	Louw [38]	2013	South Africa	65 children with ASD	A high frequency of medication, at least 24.6% of the sample, was noted in the group; additionally, around 40% of the sample reported using complementary and alternative therapies.

28	Springer [39]	2013	South Africa	58 children with pervasive developmental disorder	The study aimed at describing the demographics, history, clinical features, comorbidity, and yield of aetiological investigations in children diagnosed with a pervasive developmental disorder. The authors observed that the median age at diagnosis was 42 months. Forty percent had complex autism (dysmorphism with or without microcephaly), and 12.1% were macrocephalic. Moreover, there was high prevalence of behavioural problems (89%) and a significant proportion of the children (72.4%) were nonverbal.

29	Alant [40]	2013	South Africa	22 children with ASD from a school for individuals with ASD in South Africa	The aim of this investigation was to describe the translucency ratings of graphic symbols by a group of children with autism over repeated exposures. Although the difference between ratings on days 1 and 3 was statistically significant (medium effect size), this difference represents an overall pattern rather than significant differences on ratings of specific symbols.

30	Pileggi [41]	2013	South Africa	40 children (20 children with ASD and 20 typically developing children)	This study investigated relations among empathy and cradling bias in children diagnosed with autism spectrum disorders (ASDs). It was reported that children with ASD did not show cradling bias and it was concluded that the results support the hypothesis that leftward cradling characterized enhanced quality of caregiver-infant interaction and bonding.

31	Grinker [42]	2012	South Africa/South Korea	From South Korea: 47 participants (parents of children with ASD and teachers from regular and special education schools) From South Africa: unspecified number of clinicians, parents, daycare centre managers, traditional healers, and managers of children's homes participated	It was observed that, both in South Africa and in Korea, ASD was underdiagnosed and hardly ever reported in clinical or educational records. Additionally, both settings experienced limited resources for families of children with ASD. To be able to set up a successful programme of research, the authors heavily depended on local knowledge to solve some of the practical problems experienced.

32	Igwe [43]	2011	Nigeria	80 health workers (40 paediatric and 40 psychiatric nurses)	The authors examined knowledge about childhood ASD among paediatric and psychiatric nurses and observed a deficit in ASD knowledge among these professionals.

33	Kapp [44]	2011	South Africa	19 mothers of children with ASD	The study investigated the challenges of families with children with ASD and factors that promote resilience in these families. Some of the factors identified as promoting resilience included having a supportive family, good spousal relationship, and adequate approaches to problem-solving within the family.

34	Greeff [45]	2010	South Africa	34 parents of children with ASD	The authors investigated the factors conveying resilience in families of children with ASD and observed that higher social economic status (SES), social support, and a supportive home environment were some of the factors that contributed to resilience in the context of ASD.

35	Travis [46]	2010	South Africa	2 children with ASD	The study reports enhanced communication abilities among children with ASD who have undergone an intervention using the Picture Exchange Communication System.

36	Igwe [47]	2010	Nigeria	300 final undergraduate students	The study aimed to evaluate how much undergraduate students knew about autism in Nigeria. Results indicated that medical students were the most knowledgeable and that attendance on psychiatry and paediatric wards significantly enhanced knowledge of ASD.

37	Bakare [48]	2009	Nigeria	134 health workers	The study noted that a significant percentage of healthcare workers in Nigeria still held negative or false beliefs on the aetiology, treatability, and preventability of ASD, leading to the conclusion that part of the efforts to improve services for families of children with ASD need to focus on improving knowledge among healthcare providers.

38	Olivier [49]	2009	South Africa	8 parents of children with ASD	The study reported that some of the key challenges faced by parents of children with ASD include the denial of the diagnosis, lacking proper guidance having received the diagnosis, and a lack of adequate parenting and coping skills among other challenges.

39	Bakare [50]	2009	Nigeria	134 health workers	This study assessed the baseline knowledge about childhood ASD and opinion among Nigerian healthcare workers on availability of facilities and law caring for the needs and rights of children with childhood ASD and other developmental disorders. The workers had a moderate amount of knowledge of childhood ASD; the most salient knowledge gaps were about symptoms of obsessive behaviour and those of impairments in social interaction.

40	Bakare [51]	2008	Nigeria	50 psychiatric nurses	The study aimed to examine the psychometric properties of the Knowledge about Childhood ASD among Health Workers questionnaire. It was observed that the measure had excellent internal consistency and adequate test-retest reliability.

41	Geils [52]	2008	South Africa	One child with ASD	This case study carried out a conversational analysis of the conversations between the participant and his/her coparticipants with the aim of understanding some of the potentially useful points of intervention to enhance communication skills among children with ASD.

42	Akande [53]	2000	South Africa	3 children with ASD	This study investigated colour learning and observed the need for a highly individualized approach to teaching children with ASD since the authors observed a significant variability in the approach to learning.

43	Akande [54]	1999	South Africa	7 children with ASD	This study evaluated the efficacy of the “self-monitoring intervention” and observed that the success rates were very similar to what has been reported in other parts of the world.

44	Khan [7]	1996	Zimbabwe	18 children	The authors noted that, in the Zimbabwean context, the DSM III-R categories were supported by empirical evidence. However, they recommended the inclusion of characteristics such as abnormal responses to sensory stimuli and disturbances to cater for nonclassical autism.

45	Dhadphale [55]	1982	Kenya	3 children with infantile ASD	The study noted that there were no differences in features of childhood ASD presented by the 3 Kenyan children when compared to what has been described in the west.

46	Noach [56]	1974	South Africa	8 (4 children with ASD and 4 typically developing children)	Children with ASD were observed to present with impairments in concept formation.

47	Silver [57]	1970	South Africa	One Child with ASD	This study illustrates how operant conditioning can be used to teach a child with ASD a few words.
